# Cost Effectiveness of Quadrivalent Versus Trivalent Inactivated Influenza Vaccines for the Portuguese Elderly Population

**DOI:** 10.3390/vaccines10081285

**Published:** 2022-08-09

**Authors:** Diana Tavares, Helena Mouriño, Cristina Antón Rodríguez, Carlos Martín Saborido

**Affiliations:** 1 Faculdade de Ciências, Universidade de Lisboa, 1749-016 Lisboa, Portugal; 2 Facultad de Medicina, Universidad Francisco de Vitoria Madrid, 28223 Madrid, Spain; 3 Ministry of Health, 28014 Madrid, Spain; 4 Centro de Estudios Superiores Hygiea, 28223 Madrid, Spain

**Keywords:** influenza vaccines, decision tree, cost effectiveness analysis, one-way sensitivity analysis, probabilistic sensitivity analysis

## Abstract

Background: quadrivalent inactivated vaccine (QIV) has replaced trivalent inactivated vaccine (TIV). In Portugal, TIV is free of charge for risk groups, including older adults (≥65 years old). In its turn, QIV—which provides broader protection as it includes an additional lineage B strain—was introduced in Portugal in October 2018; only since the 2019/20 influenza season has it been provided free of charge for risk groups. This study evaluates the cost effectiveness of switching from TIV to QIV, from the National Health Service perspective, in the Portuguese elderly mainland population. Methods: A decision tree model was developed to compare TIV and QIV, based on Portuguese hospitalization data for the 2015/16 influenza season. The primary health economic outcome under consideration was the incremental cost-effectiveness ratio (ICER). In addition, one-way sensitivity analysis and probabilistic sensitivity analysis were performed. Results: the high cost of QIV (approximately three times the cost of TIV) would lead to a total increment of EUR 5,283,047, and the resulting ICER would be EUR 26,403,007/QALY, above the usual willingness-to-pay threshold. Conclusions: from the National Health Service perspective, our findings reveal that QIV is not cost effective for the Portuguese elderly population due to the high cost. If the QIV costs were the same as the TIV, then QIV would be cost effective.

## 1. Introduction

Seasonal influenza is an acute respiratory disease caused by infection with influenza viruses [1]. There are four types of seasonal influenza viruses: A, B, C, and D [1]. Only influenza A and B viruses circulate and cause seasonal epidemics of disease [1]. The infection may cause signs and symptoms like fever, cough, headache, muscle and joint pain, and weakness [1].

Influenza outbreaks are recorded every year. In temperate zones of the northern and southern hemispheres, epidemics occur during the winter, while in the tropics they occur throughout the year [2,3]. In Portugal, influenza outbreaks are characterized by significant morbidity in the general population and increased mortality rates. The high-risk groups are: the elderly (≥65 years old), patients with chronic or immunosuppressive conditions aged six months or older, pregnant women, health professionals, and other healthcare givers [4].

An influenza pandemic can appear when a new and different type of influenza A virus emerges, and it can infect humans who are not immunized [1,5]. The most recent pandemic was in 2009, caused by the A(H1N1)2009 virus, after which it became a seasonal influenza serotype [1]. In Portugal, 1189 people were hospitalized, around 10% in intensive care units, resulting in 124 deaths [6].

According to the World Health Organization (WHO), vaccination is the most effective way to prevent seasonal influenza and subsequent severe outcomes [1]. Therefore, vaccination should be administered annually to provide optimal protection. The Influenza Surveillance System in Portugal is usually activated on week 40 (October) of a given year (*n*), and lasts up to week 20 (April) of the following year (*n* + 1) [7,8]. Thus, it is recommended to administer the vaccine during autumn or winter, preferably until the end of the *n*-th year [4]. Following vaccination, the development of immunity against influenza viruses takes about two weeks [9]. However, vaccine effectiveness depends on the influenza subtypes included in the vaccines (a mismatch occurs when the viruses in the vaccine are different from the circulating viruses) and the population vaccination coverage. Unfortunately, several European countries reported a decline in vaccination coverage among older people from the 2008/09 to 2014/15 seasons. Conversely, Portugal reported an increase in vaccination uptake throughout these seasons [10]. In the 2015/16 season, influenza vaccination coverage was 50.1% in Portugal [7]. The coverage rate has continued to increase in the last few years. For instance, in the 2017/18 season, it increased to 60.8% for people aged 65 years or above [11]. We may stress that during the pandemic years caused by the SARS-CoV-2 virus, the pattern of influenza vaccination has changed fundamentally, and will not be considered here.

A Cochrane study on this topic concluded that the risk of influenza decreases from 6% to 2.5%, and the risk of influenza-like illness (ILI) reduces from 6% to 3.5%, between unvaccinated and vaccinated groups (≥65 years old) during a single season [12]. In the European Union, it is estimated that influenza vaccination prevents up to 37,000 deaths yearly [13].

There are two main types of seasonal influenza vaccines, namely inactivated influenza vaccine (IIV) and lived attenuated influenza vaccine (this one is not within the scope of the paper) [5,12]. The different IIV developed are: trivalent, trivalent adjuvanted, and quadrivalent.

Trivalent inactivated vaccine (TIV) is the most common IIV and protects against three different influenza viruses (two influenza A strains and one influenza B lineage). In some EU/EEA countries, the trivalent adjuvanted vaccine is available for older people, to empower immune response [5,14]. In Portugal, this vaccine is not available for the population.

Influenza B strain has a relevant role in the overall burden of influenza. Additionally, researchers find it very difficult every year to select the appropriate influenza B strain for inclusion in TIV; this feature has led to situations where the predominant influenza B strain for a specific season does not match the one included in the TIV for that season. Therefore, to reduce the spread and illness during the influenza season, the quadrivalent inactivated influenza vaccine (QIV) includes one more influenza B strain in addition to the one existing in TIV, which provides more comprehensive protection against influenza viruses. Quadrivalent inactivated influenza vaccine (QIV) was first approved by the FDA in 2012, and has been available since the 2014/2015 season in some EU/EEA countries; it is expected to replace TIV over time [5]. TIV is available in all EU/EEA countries, while QIV is only available in some of them. In Portugal, QIV was launched in October 2018, but only since the 2019/20 influenza season was it provided free of charge for the risk groups.

Influenza is a major global health threat affecting all countries, not only from a health perspective but also from economic and social approaches. Therefore, it is crucial to carry out economic evaluations of the influenza vaccination programs.

This work aims to estimate the cost effectiveness of switching from TIV to QIV based on the National Health Service perspective. The results are presented as incremental cost per unit of effect, described by the incremental cost-effectiveness ratio (ICER). As far as we know, no cost-effectiveness analysis of Portuguese influenza vaccination has yet been reported in the literature.

## 2. Materials and Methods

### 2.1. Key Features of the Economic Evaluation

The target population is the population of mainland Portugal aged 65 and above, because they are one of the most relevant influenza risk groups. In 2015, they represented 21% of the Portuguese population [15]. This group is usually retired, so productivity losses are not relevant. The cost-effectiveness evaluation was performed from the National Health Service (NHS) perspective, where only direct costs were considered. Thus, only the medical costs paid by the NHS are considered here.

The Influenza Surveillance System runs from week 40 of a specific year until week 20 of the following year, so the time horizon established for this work was one year [7,8]. The data under analysis refers to the 2015/16 influenza season.

As stated above, economic evaluations are performed to identify, measure, value, and compare the costs and consequences of different therapeutic alternatives for influenza vaccination. To perform the cost-effectiveness analysis, health outcomes generated by the two alternative interventions under analysis are assessed through the quality-adjusted life year (QALY). It measures the length of life years (i.e., quantity gains) and the quality of life (i.e., quality gains). The quality of life is quantified by the notion of utility: the greater the preference for a particular state, the greater its utility. Utilities incorporate in the analysis the preferences of individuals for different treatment-related outcomes. Another key measure is the concept of quality-of-life loss due to the disease (influenza, in this case); that is, disutility. It corresponds to the reverse of utility, i.e., decrease in utility due to serious health complications. These quantities are obtained by health-related quality of life (HRQoL) instruments, such as the EQ-5D family of questionnaires. Studies about utilities and disutilities for the Portuguese population are very scarce. In this work, utilities associated with different health states were obtained from a study on EQ-5D Portuguese population norms [16]. Available data were stratified by age, but unfortunately there was no information for people aged 65 or over. Therefore, the utilities for that age group were derived by interpolation (see Appendix A). Moreover, no Portuguese studies on disutilities were found for influenza. Such data were extracted from a Spanish study [17] (see Appendix A). All of these results were thus combined in a single index, QALY [18,19].

The incremental cost-effectiveness ratio (ICER) is the primary cost-effectiveness outcome used in this work [19]. To make recommendations to policymakers, ICER is usually compared with an established threshold called willingness to pay (WTP). Some decision-makers have established a willingness-to-pay (WTP) threshold that refers to the maximum ICER accepted for an intervention to be cost effective. In Portugal, there is no established cost-effectiveness threshold for health interventions. Some Portuguese authors refer to a WTP threshold of EUR 30,000/QALY [20,21], while others consider the cost-effectiveness threshold to be twice the gross domestic product (GDP) per capita or gross national income (GNI) per capita [21,22]. In 2015, the Portuguese GNI per capita was EUR 16,887.36, resulting in a ceiling ratio of approximately EUR 34,000 (twice the GNI) [23,24]. In this paper, the ICER will be used to provide information on the extra amount necessary to pay in order to gain an extra QALY, when the most effective alternative is chosen [19,25].

### 2.2. Decision Tree

The cost-effectiveness analysis was based on a decision tree model, a very powerful tool in this context because it incorporates probabilities of the outcomes, resource costs, vaccination coverage rate, prices of the vaccines, efficacy, utilities, and disutilities. It consists of a series of pathways representing possible prognoses for each alternative therapy under evaluation. A static model was used, and no herd effects of vaccination were evaluated.

**Figure 1:**
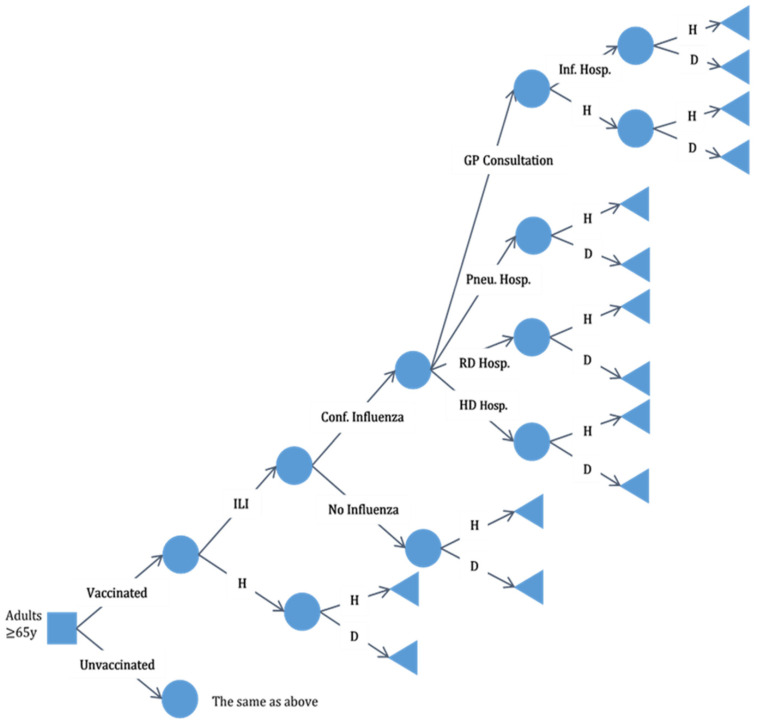
Decision tree for the cost-effectiveness analysis. Branch for the vaccinated people aged ≥65 years old. Abbreviations: ILI, influenza-like illness; H, healthy; D, death; conf. influenza, confirmed influenza; GP consultation, general practitioner consultation; pneu. hosp., hospitalization due to pneumonia; RD hosp., hospitalization due to respiratory disease; HD hosp., hospitalization due to heart disease; inf. hosp., hospitalization due to influenza.

One decision tree model was developed for each alternative therapy (TIV and QIV). For convenience, each decision tree was split into two different diagrams referring to vaccinated and unvaccinated people. To illustrate this, Figure 1 displays the branch of the decision tree for vaccinated individuals.

We assumed that influenza confirmation (through diagnostic tests) was carried out in the context of general practitioner (GP) consultation, pneumonia hospitalization, respiratory disease hospitalization, or heart disease hospitalization. Although it is known that influenza may be diagnosed in many other situations, the diseases described above are usually associated with complications due to influenza in the elderly. It should be noted that the influenza event (as a secondary diagnosis) may have occurred before or after hospital admission in which the principal diagnosis was pneumonia, respiratory disease, or heart disease. It is important to note that the “healthy” state is assumed to include all individuals who did not die, regardless of their health conditions.

### 2.3. Input Parameters and Computation of the Probabilities

The decision tree is powered by different types of parameters. A detailed description of each of these measures is provided in Appendix B (it includes Table A1). A summary of the base case values for the parameters under consideration is displayed in Table 1. The computation of the different parameters involved in the decision tree pathways for the TIV—probabilities, costs, and QALY—are provided in the Figure S1 and Table S1. Similar calculations are made for the QIV scenario.

### 2.4. Scenario Analysis

The most common ways to characterize parameter uncertainty are: one-way sensitivity analysis (OWSA) and probabilistic sensitivity analysis (PSA) [19,25].

OWSA consists of varying the point estimates of the input parameters one at a time within a plausible range of values and assessing the impact on model outcomes [19,25], which corresponds to the ICER. In this work, we performed OWSA; when the confidence interval (CI) for a specific parameter was determined from the literature, these limits were used as the OWSA range. For parameters with no CI available, the usual range of ±20% was used to assess the model’s sensitivity to parameters’ variation. A tornado diagram was used to report OWSA results.

A scenario analysis of the vaccination coverage rate, TIV effectiveness, and QIV effectiveness variation was carried out. The cost of the quadrivalent vaccine was identified as a critical parameter in the analysis; therefore, it was also studied in detail [45]. For the vaccination coverage rate and QIV cost, a sequence of parameter values was generated with an increment of 0.05. For TIV and QIV effectiveness, we applied a thinner grid of points using an increment of 0.005 to analyze in detail the relationship between each variable and ICER.

Instead of representing the parameters as single-point estimates as in OWSA, in PSA parameters are represented by random variables following a particular distribution. Table S1 displays the base case values used for each input parameter of the decision tree models. It also shows the probability distribution (with information on location, shape, and scale, when appropriate) used for each parameter under consideration. A total of 1000 Monte Carlo (MC) simulations were performed to carry out PSA.

## 3. Results

### 3.1. Base Case Analysis

Table 2 summarizes the base case results of shifting from TIV to QIV in the elderly, based on the 2015/16 influenza season data. Results show a difference in confirmed influenza cases of approximately 37. As a result, about 36 GP consultations could have been averted and, therefore, EUR 1103 could have been saved. In addition, five hospitalizations, including hospitalizations due to influenza, pneumonia, respiratory disease, and heart disease, could have been averted and one life saved.

Regarding hospitalization and death costs, caution must be taken to interpret the results in Table 2. The cost of death for an inpatient includes all hospitalization costs; in the same way, the hospitalization costs also include the costs of people who died. Thus, EUR 15,625 could have been saved in hospitalizations: EUR 9275 in hospitalizations due to influenza; EUR 1705 in hospitalizations due to pneumonia; EUR 1285 in hospitalizations due to respiratory disease; and EUR 3361 in hospitalizations due to heart disease. From that value, EUR 3694 are related to hospitalizations of patients who died (Table 2).

In what concerns the number of vaccinated people (mentioned in Table 2 as “vaccine doses”), the value is the same for both strategies. It was an expected result because the vaccination coverage rate applied to TIV and QIV models was the same (the paper’s focus is to evaluate the cost effectiveness of shifting from TIV to QIV). However, there is a difference in the expected costs computed from the two strategies because they depend on the cost of each vaccine. A total of 1,035,895 vaccine doses were expected to have been administered to individuals aged ≥65 years old in the 2015/16 season. For the TIV strategy, this represented spending EUR 2,668,465, while for QIV corresponds to EUR 7,968,557; that is, shifting from TIV to QIV would result in an additional cost of EUR 5,300,092 (Table 2).

The expected cost difference between TIV and QIV is EUR 5,283,047, while the difference in effects (QALYs) is expected to be 0.20. These values result in a base case ICER of EUR 26,403,007/QALY (Table 2). The plot of the cost differences and effects on the cost-effectiveness plane shows that the value under consideration is in the first quadrant. Thus, for a cost-effectiveness threshold of EUR 34 000/QALY (twice the GNI per capita), the new vaccine would not be cost effective.

### 3.2. One-Way Sensitivity Analysis

**Figure 2:**
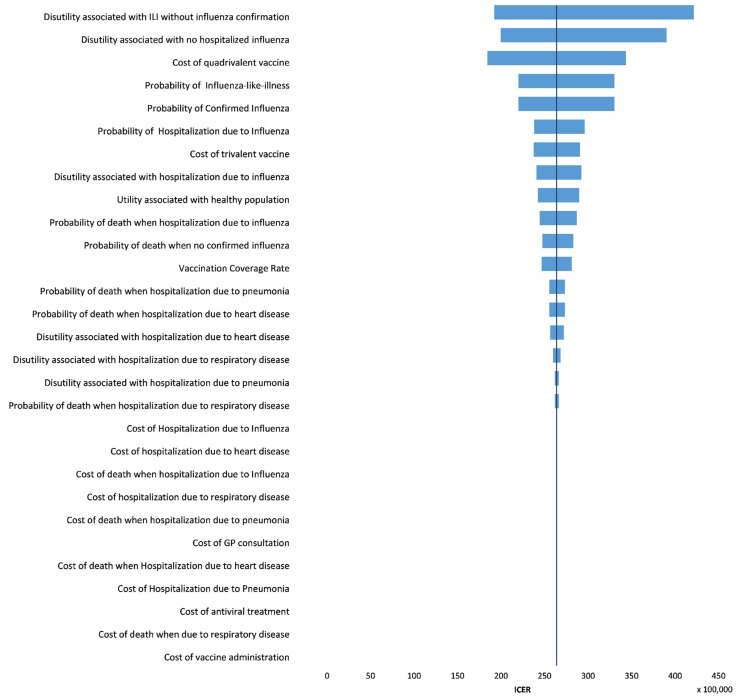
One-way sensitivity analysis: tornado diagram. The horizontal bars represent the estimated changes in the ICER for the range of variation of each parameter under consideration. The parameters are sorted by descending order of importance. The vertical line corresponds to the base case result.

Figure 2 summarizes the one-way sensitivity analysis using the tornado diagram.

Figure 2 reveals that the disutility associated with ILI when no confirmed influenza, and the disutility associated with no hospitalized influenza have the highest impact on the ICER. For example, if the first parameter mentioned above assumes the value of 0.007 (lower bound), it results in an ICER of EUR 19,219,556/QALY; and the value of 0.011 (upper bound) results in an ICER of EUR 42,160,989/QALY. This feature can be explained by the increase in incremental QALYs, yielded by decreasing this parameter, resulting in a reduced ICER and vice-versa. In contrast, the variation of the second parameter within the 95% CI (0.007, 0.011) produced ICERs of EUR 39,019,821/QALY and EUR 19,951,738/QALY, respectively. Thus, while the pathway corresponding to vaccinated patients with ILI but not confirmed influenza is more populated in the QIV model than in TIV, the same is not valid for the pathway referring to vaccinated patients with no hospitalized influenza. Thus, a reduction in disutility associated with no hospitalized influenza would decrease incremental QALYs and increase ICER.

**Figure 3:**
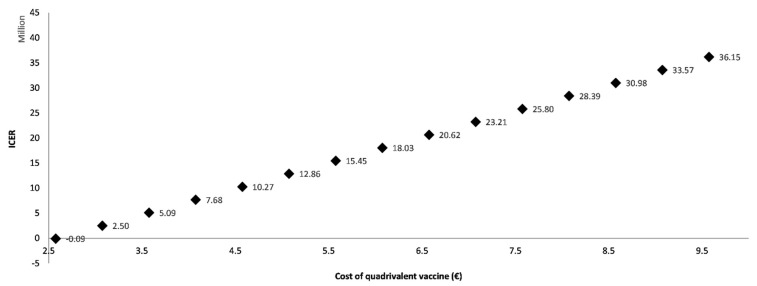
One-way sensitivity analysis: graphical plot of ICER versus cost of QIV. Variation between EUR 2.576 (the cost of TIV) and EUR 9.756 with increments of EUR 0.05.

The cost of the quadrivalent vaccine is the third parameter appearing in the tornado diagram (Figure 2). The variation of the cost of the quadrivalent vaccine within a ±20% range results in an ICER of EUR 18,438,140/QALY (lower bound) and EUR 34,367,874/QALY (upper bound), respectively. The high impact of QIV’s cost on ICER was expected if attention is paid to the difference in costs of QIV and TIV vaccine doses (Table 2). The cost of QIV’s vaccines doses is largely above the cost of TIV’s vaccine doses. Further analysis was performed, varying the QIV cost within a broader range (Figure 3). For a QIV cost equal to TIV cost, an ICER of EUR −85,182/QALY was obtained, meaning that shifting from TIV to QIV would be cost saving, as expected. However, a value higher than EUR 2.576 would quickly lead to being not cost effective.

The probability of ILI and the probability of confirmed influenza also have a high impact on ICER (Figure 2). Varying these parameters by 20% downward and upward produced ICERs of EUR 33,025,054/QALY and EUR 21,988,309/QALY, respectively. Thus, as the probability of ILI and/or the probability of confirmed influenza increases, the ICER decreases.

One-way sensitivity analyses of the effectiveness of QIV and TIV are displayed in Figure S2 and S3, respectively. If the TIV effectiveness is equal to QIV effectiveness, the ICER is not defined, as it results in a difference in effects equal to zero and, therefore, a division by zero, and a vertical asymptote at 59.91% is shown (Figure S2). As TIV effectiveness approaches 59.91% from the left, the ICER tends toward positive infinity. Otherwise, as TIV effectiveness approaches 59.91% from the right, the ICER tends toward negative infinity. Such behavior is explained by the difference in effects, close to zero, when TIV and QIV effectiveness are close. A negative ICER is produced for TIV effectiveness values higher than QIV effectiveness as the incremental QALYs value is negative. Similarly, Figure S3 shows a vertical asymptote when QIV effectiveness equals 58%; ICER tends to infinity when it approaches the vertical asymptote from the right. Finally, the higher the effectiveness of quadrivalent vaccines (>58%), the lower the ICER.

The vaccination coverage rate is a very effective preventive measure for influenza. For this reason, a detailed study was also performed for this parameter (Figure S4). A higher coverage rate would result in decreased ICER. As the World Health Assembly recommended, for a coverage rate of 75%, EUR 21,010,680/QALY would be obtained, corresponding to a reduction of 20.42%.

### 3.3. Probabilistic Sensitivity Analysis

Probabilistic sensitivity analysis allows one to evaluate the robustness of the base case results. To attain this goal, a probability distribution was assigned to each parameter of the model to reflect parameter uncertainty due to sampling errors. Parameters values were then sampled 1000 times; expected costs and QALY were recorded from the 1000 MC simulations. Finally, some empirical statistics such as mean, standard deviation, and confidence intervals were computed.

In general, the results from PSA (Table S2) are similar to those obtained from the base case analysis (Table 2). The estimated mean costs difference between QIV and TIV is EUR 5,293,456 with an interval estimate of (EUR 4,375,217; EUR 6,088,637), while the estimated mean effect difference corresponds to 0.20 QALYs with an interval varying from 0.06 to 0.43 QALYs. On its turn, mean ICER is estimated at EUR 34,501,793/QALY (EUR 13,052,665/QALY; EUR 87,418,632/QALY). This value is about 31% higher than in the base case result.

Figure S5 displays the scatterplot of the 1000 MC simulations to compare TIV and QIV, that is, the graphical representation of costs against QALYs. The base case results are also shown. QALY of both interventions varies within a similar range of values (it varies between 1,155,190 and 1,375,507). On the other hand, the difference between TIV and QIV in terms of costs is very well delimited, with QIV being costlier than TIV (QIV: mean = EUR 13,323,148; SD = EUR 987,347; TIV: mean = EUR 8,029,692; SD = EUR 548,739).

**Figure 4:**
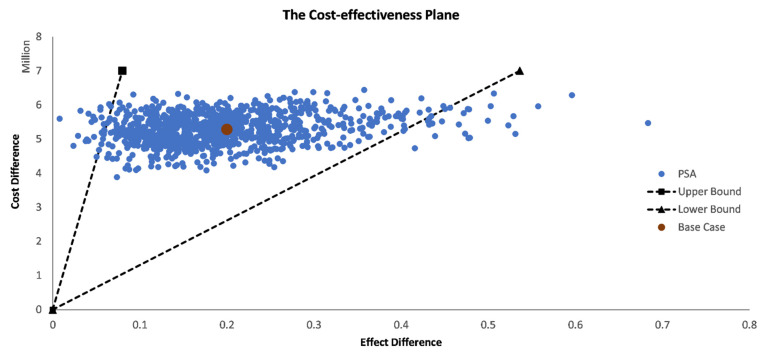
Probabilistic sensitivity analysis for TIV and QIV. Cost-effectiveness plane based on data from the 2015/16 influenza season. The orange dot represents the base case. For each simulation, the incremental effect is represented in the *x*-axis, whereas the incremental cost is displayed in the *y*-axis, in comparison to the base case (scatterplot). The 95% confidence interval is displayed by the black dashed lines.

Figure 4 shows the results of the simulations in a cost-effectiveness plane, where the incremental costs are plotted against the incremental effects. According to Figure 4, the cost-effectiveness results are robustly located in the first quadrant of the cost-effectiveness plane, and all simulated ICERs are higher than CE thresholds previously established.

## 4. Discussion

To the best of our knowledge, this is one of the first published studies to evaluate seasonal influenza vaccination cost effectiveness in Portugal. Only a few papers studied the burden of disease and vaccination effectiveness in the Portuguese population; for instance, the international I-MOVE+ project [46,47,48,49,50].

We evaluated the cost effectiveness of switching from TIV to QIV based on the 2015/16 influenza season data. Base case results revealed that the universal substitution of TIV with QIV would result in an ICER of EUR 26,403,007 per QALY gained. As such, for the cost-effectiveness thresholds of EUR 30,000/QALY or EUR 34,000/QALY, QIV is not cost effective. Probabilistic sensitivity analysis enhanced the robustness of the base case results. The ICER is much higher than any possible ceiling ratio established by NHS. Such results are comparable to those obtained for the first year of QIV administration for the Hong Kong elderly (≥80 years old) population [51]. The need for a longer time horizon is here emphasized.

In most international papers [45,52,53,54,55,56,57,58,59,60], QIV is usually considered cost effective compared with TIV. As cited above, one of the reasons might be using a longer time horizon to calculate QIV effectiveness, which allowed researchers to posit a few seasons that match the TIV with the circulating strains and, therefore, low mismatching (see Appendix B). Another reason might be the cost of QIV, which in this study is about three times the TIV. Regarding the quadrivalent vaccine, its cost is 200% higher than TIV. However, in the literature, the additional cost of QIV is rarely higher than 100% of the TIV cost [52,54,55,56], which might be one of the reasons why QIV is cost effective in some studies. Therefore, to be cost effective, the cost of QIV would need to be the same as TIV in our case. It is important to emphasize that QIV has been recently developed and marketed, which has led to a high price. In the near future, manufacturers will likely scale up the production of QIV, resulting in price reduction. This change in QIV’s cost might affect the results from the cost-effectiveness analysis developed in this article, and QIV is likely to be cost effective in the future due to a decrease in its cost.

In the literature, the probability of influenza-like illness and the probability of confirmed influenza was found to be higher than those used here. For example, Capri et al. [60] applied a probability of influenza of 6.4%, resulting from the product of ILI attack rate (16.8%) by influenza virus isolation rate (32.1%), while Van Bellinghen et al. [55] used a probability of symptomatic influenza infection of 6.17%. In our study, the probability of confirmed influenza was derived from a Portuguese report on influenza surveillance, and the probability of ILI was determined from other input values. It is comparable to the value reported by INSA for the 2015/16 influenza season [31].

One-way sensitivity analysis demonstrated that disutility associated with ILI, disutility associated with no hospitalized influenza, cost of quadrivalent vaccine, probabilities of influenza-like-illness and of confirmed influenza are the five most influential variables on ICER. The most relevant input parameters obtained from the OWSA are in line with other OWSA results in the literature [52,55,56]. Considering the PSA, the main results from the cost-effectiveness plane, interval estimates for the parameters, and the cost-effectiveness acceptability curve are in line with the base case conclusions and confirm their robustness.

A limitation of our study is that the model is static in a way that herd effects of immunization are not considered, and only direct protection is captured. Thus, vaccination’s impact on the disease’s burden might be underestimated. In the future, a dynamic transmission model might be a possible approach to account for the impact of vaccination on influenza transmission as well as the effect of other influential variables [45,56]. For instance, Van Bellinghen et al. [56] used a lifetime, multi-cohort, static Markov model to compare the potential effects of TIV and QIV on the disease burden of influenza in the UK from the NHS’s perspective.

Side effects of vaccination were not included in this study, but QIV safety is assumed to be comparable to that of TIV, and side effects are assumed to be mild and temporary. Thus, no impact is expected on the ICER [56].

Further investigation is required to fully understand the cost effectiveness of QIV versus TIV in Portugal. Future research might explore the cost effectiveness of influenza vaccination for the entire population, and not only focusing on the elderly population. For instance, Boer et al. [45] applied a dynamic transmission model to estimate age-stratified numbers of symptomatic influenza B cases under TIV and QIV strategies. Alternatively, we could use a multi-scale network-based model of contagious dynamics to describe the spread of influenza in the population, and afterwards develop a cost-effectiveness analysis. This model would also allow one to evaluate the impact of the vaccination strategy [61].

In summary, this study contributes to understanding the impact of annual influenza epidemics on health economics and public health in Portugal.

## Figures and Tables

**Table 1:** Input parameters for the decision tree: base case values.

Input Parameters	Base Case	Source
Population	2,067,654	[26]
Vaccination coverage rate	0.501	[7]
Trivalent vaccine effectiveness	0.580	[12]
Quadrivalent vaccine effectiveness	0.599	[27,28,29,30,31,32,33,34,35]
Probability of influenza-like illness	0.007	[7,12,26,31,36]
Probability of confirmed influenza	0.278	[31]
Probability of GP consultation	0.952	[31,36]
Probability of hospitalization due to influenza	0.091	[37]
Probability of death when hosp. influenza	0.079	[37]
Probability of hospitalization due to pneumonia	0.008	[37]
Probability of death due to hosp. pneumonia	0.381	[37]
Probability of hospitalization due to respiratory disease	0.014	[37]
Probability of death when hosp. RD	0.050	[37]
Probability of hospitalization due to heart disease	0.026	[37]
Probability of death when hosp. HD	0.110	[37]
Probability of death when no confirmed influenza	0.043	[26,38]
Cost of GP consultation	31.000	[39]
Cost of hospitalization due to influenza	2644.011	[37,39]
Cost of death when hospitalization due to influenza	6364.597	[37,39]
Cost of hospitalization due to pneumonia	2906.749	[37,39]
Cost of death when hospitalization due to pneumonia	11,298.219	[37,39]
Cost of hospitalization due to respiratory disease	2534.841	[37,39]
Cost of death when hospitalization due to RD	1363.460	[37,39]
Cost of hospitalization due to heart disease	2978.842	[37,39]
Cost of death when hospitalization due to HD	7785.951	[37,39]
Cost of antiviral treatment	18.300	[40,41]
Cost of trivalent vaccine	2.576	[42]
Cost of quadrivalent vaccine	7.692	[43,44]
Cost of vaccine administration	3.700	[39]
Disutility associated with ILI without influenza confirmation	0.009	[17]
Disutility associated with no hospitalized influenza	0.009	[17]
Disutility associated with hospitalization due to influenza	0.031	[17]
Disutility associated with hospitalization due to pneumonia	0.031	[17]
Disutility associated with hospitalization due to respiratory disease	0.031	[17]
Disutility associated with hospitalization due to heart disease	0.031	[17]
Utility associated with healthy population	0.625	[16,26]

**Table 2:** Results of the cost-effectiveness evaluation comparing TIV and QIV (base case).

Parameter	TIV	QIV	Difference (QIV-TIV)
Events			
GP consultations	2631.35	2595.77	−35.58
Hospitalizations due to influenza	239.00	235.77	−3.23
Deaths due to influenza hospitalization	19.00	18.74	−0.26
Hospitalizations due to pneumonia	21.00	20.72	−0.28
Deaths due to pneumonia hospitalization	8.00	7.89	−0.11
Hospitalizations due to RD	40.00	39.46	−0.54
Deaths due to RD hospitalization	2.00	1.97	−0.03
Hospitalizations due to HD	73.00	72.01	−0.99
Deaths due to HD hospitalization	8.00	7.89	−0.11
Vaccine doses	1,035,895	1,035,895	0.00
Costs			
GP consultations	EUR 81,572	EUR 80,469	EUR −1103
Hospitalizations due to influenza	EUR 710,464	EUR 701,189	EUR −9275
Deaths due to influenza hospitalization	EUR 121,552	EUR 119,934	EUR −1617
Hospitalizations due to pneumonia	EUR 128,213	EUR 126,508	EUR −1705
Deaths due to pneumonia hospitalization	EUR 90,401	EUR 89,189	EUR −1211
Hospitalizations due to RD	EUR 99,125	EUR 97,841	EUR −1285
Deaths due to RD hospitalization	EUR 2731	EUR 2697	EUR −34
Hospitalizations due to HD	EUR 256,048	EUR 252,687	EUR −3361
Deaths due to HD Hospitalization	EUR 62,303	EUR 61,471	EUR −831
Vaccine doses	EUR 2,668,465	EUR 7,968,557	EUR 5,300,092
Total	EUR 8,018,570	EUR 13,301,618	EUR 5,283,047
QALYs (Total)	1,265,456.95	1,265,457.15	0.20
ICER (EUR/QALY)			26,403,007

## Data Availability

The data for the work were partially derived from the published literature. This published literature is cited in the manuscript and the complete reference is provided in the Reference list. Additionally, the authors used the Portuguese database BDMH (Base de Dados de Morbilidade Hospitalar), ACSS.

## Supplementary Materials

The following are available online at https://www.mdpi.com/article/10.3390/vaccines10081285/s1, Figure S1: Probability, cost and QALY calculations by decision tree pathway for individuals aged ≥65 years old who received TIV; Table S1: Description of the variables that take part of Figure S1; Figure S2: One-way sensitivity analysis of TIV effectiveness. Variation between 34% and 74% with increments of 0.005; Figure S3: One-way sensitivity analysis of QIV effectiveness. Variation between 58% (TIV effectiveness) and 100% with increments of 0.005; Figure S4: One-way sensitivity analysis of vaccination coverage rate. Variation between 0% and 100% with increments of 0.05; Figure S5: Probabilistic sensitivity analysis for TIV and QIV. Scatter plot based on the 2015/16 influenza season; Table S2: Input parameters. Base case value; SD; parameters of the probability distribution; Table S3: Probabilistic sensitivity analysis. Assessing the 2015/16 influenza season-related uncertainty.

## Appendix A

The cost-effectiveness analysis carried out in this paper relies on the computation of QALY, which has become widely used in this type of study. It is thus assumed that the major objective is to maximize health or health improvement across the population, subject to resource constraints. The greatest advantage of QALY is the combination of changes in morbidity and mortality in a single indicator. The core concept of the conventional QALY is grounded in decision science and expected utility theory [62]. In order to estimate the value attributed to the different health states by the Portuguese individuals (that is, to estimate the utilities), we used the health questionnaires developed by EuroQol, with special emphasis on EQ-5D. Thus, the health utilities assigned to the different state of health under consideration are based on published studies that applied the EQ-5D instrument.

There are few studies published in Portugal about QALY weights calculation. Utilities associated with a healthy state were obtained from a study on EQ-5D Portuguese population norms [16]. Data were stratified by age, but there was no stratum for people aged 65 or over. In the same way, no studies were found about disutilities associated with influenza. Such data were extracted from Hollmann et al. [17], where QALYs losses due to no hospitalized influenza and ILI without influenza confirmation were derived from individual outpatients QALY losses. On the other hand, disutilities associated with hospitalizations due to influenza, pneumonia, respiratory disease and heart disease were derived from individual inpatient QALY losses [17], based on the EQ-5D system.

QALYs were calculated annually, as the time horizon chosen was one year. In the pathways ending in the “death” state, the utility associated with a healthy state was divided by two. This is done because no time of death is known, so it is assumed to have occurred in the middle of the year.

## Appendix B

### Appendix B.1. Model Input Parameters

Most of the data were extracted from the Portuguese Hospital Morbidity Database (HMDB) [37], which is owned by the Central Administration of the Health System Portugal. It gathers information—on the patient level—about inpatient and outpatient administrative data, as well as hospital episode data. The data extracted from the HMDB consist of admissions of patients aged 65 or above, with a principal diagnosis of influenza, pneumonia, respiratory disease, or heart disease in 2015. For hospitalizations due to pneumonia, respiratory disease, and heart disease, only subjects who experienced an episode of influenza as a secondary diagnosis were considered.

The total population aged ≥65 year-old was derived from an annual indicator released by Statistics Portugal, named resident population by place of residence (NUTS—2013), sex and age [26]. Data on vaccination coverage in 2015/2016 season were taken from the following report published by the (Portuguese) National Health Institute Dr. Ricardo Jorge (INSA): Vacinação antigripal da população portuguesa na época 2015/2016 [7].

Trivalent inactivated vaccine effectiveness was established at 58%, according to Demicheli et al. [12]. It was calculated as 1-RR, where RR (risk ratio) corresponds to the ratio between the proportions of patients who developed influenza in vaccinated and unvaccinated patients. The latter are patients who received placebo [12]. Quadrivalent inactivated vaccine effectiveness was estimated through the study of the proportion of circulating B strains not included in TIV across several seasons, summarized in Table A1. The methodology was adapted from Petri and Ruiz-Aragón [34]. The data were retrieved from the Portuguese National Influenza Surveillance Programme (NISP) annual reports for seasons from 2010/11 to 2016/17 [27,28,29,30,31,32,33]. Data related to season 2017/18 were derived from the weekly report on influenza surveillance published by INSA [35]. On average, 8.69% of the circulating strains correspond to mismatched TIV strains. The next step was to obtain the relative gain in effectiveness of using QIV instead of TIV, which was calculated as 8.69% × 0.38 = 3.30%, where 0.38 indicates the mean reduction in vaccine effectiveness due to B lineage mismatch [34].

**Table A1:** Proportion of B lineage influenza virus not included in the seasonal trivalent vaccines from 2010/11 to 2017/18 influenza seasons.

Season	Proportion of Influenza B	Proportion of B/Victoria	Proportion of B/Yamagata	B—lineage in TIV	Proportion of B Lineage Mismatch	SE	Source
2010/11	43.20%	42.70%	0.50%	Victoria	0.50%	0.00022	[28]
2011/12	2.30%	0.00%	2.30%	Victoria	2.30%	0.00058	[29]
2012/13	51.30%	1.80%	49.50%	Yamagata	1.80%	0.00038	[30]
2013/14	0.80%	0.10%	0.70%	Yamagata	0.10%	0.00011	[31]
2014/15	0.36%	0.00%	0.36%	Yamagata	0.00%	0.00000	[32]
2015/16	8.30%	7.80%	0.50%	Yamagata	7.80%	0.00084	[33]
2016/17	0.20%	0.20%	0.00%	Victoria	0.00%	0.00000	[34]
2017/18	66.00%	9.00%	57.00%	Victoria	57.00%	0.00354	[35]
2010/11–2017/18	21.56%				8.69%	0.00213	

### Appendix B.2. Computation of the Probabilities

The probability of confirmed influenza for people aged ≥65 years was based on the NISP annual report for 2015/2016 season [31]. Data on *GP* consultations were extracted from the National Health Service online platform named Seasonal Health [36]. The number of patients (≥65 y) who attended the *GP* consultation in the context of confirmed influenza, denoted by $N_{GP\;consultation}$, is provided by:

$$N_{GP\;consultation}=N_{TotalGP\;consultation}\times p_{\geq 65y|ILI}\times p_{Influenza}$$

where $N_{TotalGP\;consultation}$ represents the total number of *GP* consultations for influenza; that is, the sum of *GP* consultations per week, from week 40 of 2015 to week 20 of 2016; $p_{\geq 65y|ILI}$ is the population proportion of patients aged 65 years or above who have *ILI*, obtained from the NISP annual report for the 2015/2016 season [31]; $p_{Influenza}$ is the population proportion of patients with confirmed influenza obtained from the same report [31].

The probability of *ILI*, denoted by $p_{ILI}$, thus takes the form:

$$P(ILI)=\frac{N_{GP\;consultations}}{N_{\geq 65y}\times VC\times P(Influenza)\times \left(1-TIV_{effect}\right)+N_{\geq 65y}\times \left(1-VC\right)\times P(Influenza)}$$

where $N_{\geq 65y}$ is the total number of individuals aged 65 and over in 2015, described in the section “Input Parameters”; *VC* (stands for vaccination coverage rate), and $TIV_{effect}$ (represents *TIV* effectiveness) are also described in “Input Parameters.” It should be noted that *TIV* effectiveness was considered in the calculation of *ILI*, because in the 2015/16 influenza season only the *TIV* vaccine was administered.

Probabilities of hospitalization due to influenza, pneumonia, respiratory disease, and heart disease, as well as probabilities of death when hospitalized due to such conditions were obtained from the HMDB [37]. Probability of death without influenza confirmed was based on the following annual indicators developed by Statistics Portugal: resident population by place of residence (NUTS—2013), sex and age [26]; and number of deaths by place of residence (NUTS—2013), sex and age [38].

### Appendix B.3. Costs

Cost of GP consultation and cost of vaccine administration were established by the Portuguese government and can be found in the Ordinance No. 234/2015 of 7th August, issued by the State Secretary for Health. All costs related to hospitalizations were calculated from the Portuguese Hospital Morbidity Database (HMDB) [37]. The “episode cost” is obtained by the product of “relative weight,” “equivalent patient,” and “base price.” The first term is available on the price table defined by the NHS for 2015 [39]; “equivalent patient” allows adjusting the “episode cost” according to the episode length, which begins with inpatient admission and ends with inpatient discharge; “inpatient episode” may be classified as normal, short, or long stay according to the interquartile range of the respective diagnosis-related group (DRG) (i.e., normal if it falls within the range, short if it is less than the lower bound, and long if it is greater than the upper bound). Thus, each inpatient episode is converted into an equivalent patient considering the interquartile range defined for each DRG and the duration of the inpatient episode. Finally, the inpatient base price was defined by the Central Administration of the Health System Portugal and it corresponds to EUR 2285 [39]. Input costs of hospitalizations and deaths correspond to the average costs. It is important to note that the cost of death when hospitalized includes all costs related to the last hospitalization of the patient, so the cost of hospitalization is not considered in the pathways of the decision tree that leads to the “death” state.

Cost of antiviral treatment (AT) and cost of TIV for NHS were retrieved from online public contracts (available on the online platform BASE: Contratos Públicos Online [40]). According to Orientation N° 007/2015 from the Regulatory Decree N° 14/2012 of the 26 January [41], article 2, paragraph 2(a), the administration of 75 mg of oseltamivir was recommended twice daily for 5 days. As a result, the total cost of 10 capsules was considered. Cost of AT was considered in all pathways with ILI. It is recommended to be administered at the earliest stage, preferably in the first 48 h after the onset of the symptoms, and laboratory confirmation is not needed during influenza activity season. It was assumed that the cost of hospitalization and cost of death when hospitalized include the cost of antiviral treatment, so it was not added in such pathways.

Cost of TIV in 2015 was not available, so it was assumed to be the same as in 2016 [42]. As QIV was recently reimbursed by the NHS, the model was updated with QIV cost from the 2019/2020 season, obtained from the online platform [43]. It was applied at a discount rate according to the average growth rate of the consumer price indexes from 2016 to 2019, which was retrieved from Statistics Portugal [44].
