# Supplementary material for: Cost Effectiveness of Quadrivalent Versus Trivalent Inactivated Influenza Vaccines for the Portuguese Elderly Population
Source: Vaccines (Basel). 2022 Aug 9;10(8):1285. doi: 10.3390/vaccines10081285 (PMC9416623; doi:10.3390/vaccines10081285)
Supplement: Supplementary file 1 [file vaccines-10-01285-s001.zip › Table S1.pdf]

Table S1. Description of the variables that take part of Figure S1.

Code	Description	Code	Description
p_ili	Probability of Influenza-like-illness	c_death_hosp_RD	Cost of death when Hospitalization due to RD
p_conf_ili	Probability of Confirmed Influenza	c_hosp_HD	Cost of Hospitalization due to Heart Disease
p_gp	Probability of GP Consultation	c_death_hosp_HD	Cost of death when Hospitalization due to HD
p_hosp_inf	Probability of hospitalization due to Influenza	c_death	Cost of death
p_death_inf	Probability of death when Hosp. Influenza	c_ant_treat	Cost of antiviral treatment
p_hosp_pneu	Probability of hospitalization due to Pneumonia	c_tiv	Cost of trivalent vaccine
p_death_pneu	Probability of death due to Hosp. Pneumonia	c_qiv	Cost of quadrivalent vaccine
p_hosp_RD	Probability of hospitalization due to Respiratory Disease	c_vac_admin	Cost of vaccine administration
p_death_RD	Probability of death when Hosp. RD	u_ili_no_inf	Disutility associated with ILI without influenza confirmation
p_hosp_HD	Probability of hospitalization due to Heart Disease	u_inf_no_hosp	Disutility associated with no hospitalized influenza
p_death_HD	Probability of death when Hosp. HD	u_hosp_inf	Disutility associated with hospitalization due to influenza
p_death_no_inf	Probability of death when no confirmed influenza	u_hosp_pneu	Disutility associated with hospitalization due to pneumonia
c_ili_no_inf	Cost of ILI without influenza confirmation	u_hosp_RD	Disutility associated with hospitalization due to respiratory disease
c_GP	Cost of GP consultation	u_hosp_HD	Disutility associated with hospitalization due to heart disease
c_hosp_inf	Cost of Hospitalization due to Influenza	u_healthy	Utility associated with healthy population

c_death_inf	Cost of death when Hosp. Influenza	Pop	Population
c_hosp_pneu	Cost of Hospitalization due to Pneumonia	Coverage	Vaccination Coverage Rate
c_death_hosp_pneu	Cost of death when Hospitalization due to Pneumonia	tiv_effect	Trivalent Vaccine Effectiveness
c_hosp_RD	Cost of Hospitalization due to Respiratory Disease	qiv_effect	Quadrivalent Vaccine Effectiveness
