# Supplementary material for: Cost Effectiveness of Quadrivalent Versus Trivalent Inactivated Influenza Vaccines for the Portuguese Elderly Population
Source: Vaccines (Basel). 2022 Aug 9;10(8):1285. doi: 10.3390/vaccines10081285 (PMC9416623; doi:10.3390/vaccines10081285)
Supplement: Supplementary file 1 [file vaccines-10-01285-s001.zip › Table S2.pdf]

Table S2. Input parameters. Base case value; SD; parameters of the probability distribution.

Input Parameters	Base Case	SD	Distribution	Parameter 1	Parameter 2
Probability of Confirmed Influenza	0.278	0.037	Beta	39.72	103.28
Probability of being ≥65years when ILI	0.130	0.0095	Beta	162.87	1089.13
Disutility associated with ILI without influenza confirmation	0.009	0.0010	Gamma	77.79	0.00012
Disutility associated with no hospitalized influenza	0.009	0.0010	Gamma	77.79	0.00012
Disutility associated with hospitalization due to influenza	0.031	0.0031	Gamma	102.55	0.00030
Disutility associated with hospitalization due to pneumonia	0.031	0.0031	Gamma	102.55	0.00030
Disutility associated with hospitalization due to respiratory disease	0.031	0.0031	Gamma	102.55	0.00030
Disutility associated with hospitalization due to heart disease	0.031	0.0031	Gamma	102.55	0.00030
Utility associated with healthy population	0.625	0.0163	Gamma	525.41	0.00071
Vaccination coverage rate	0.501	0.041	Beta	74.68	74.38
Proportion of B lineage viruses not included in TIV	0.087	0.002	Beta	15.77	165.81
Relative Risk (1-Trivalent Vaccine Effectiveness)	0.421	0.228	Lognormal	-0.865	0.228

SD: Standard Deviation
