# Supplementary material for: Cost Effectiveness of Quadrivalent Versus Trivalent Inactivated Influenza Vaccines for the Portuguese Elderly Population
Source: Vaccines (Basel). 2022 Aug 9;10(8):1285. doi: 10.3390/vaccines10081285 (PMC9416623; doi:10.3390/vaccines10081285)
Supplement: Supplementary file 1 [file vaccines-10-01285-s001.zip › Table S3.pdf]

Table S3. Probabilistic sensitivity analysis. Assessing the 2015/16 influenza season-related uncertainty.

Parameter	TIV	QIV	Difference (QIV-TIV)
Events			
GP Consultations	2,636.73	2,602.02	-34.71
Hospitalizations due to Influenza	239.00	235.85	-3.15
Deaths due to Influenza Hospitalization	19.00	18.75	-0.25
Hospitalizations due to Pneumonia	21.00	20.72	-0.28
Deaths due to Pneumonia Hospitalization	8.00	7.89	-0.11
Hospitalizations due to RD	40.00	39.47	-0.53
Deaths due to RD Hospitalization	2.00	1.97	-0.03
Hospitalizations due to HD	73.00	72.04	-0.96
Deaths due to HD Hospitalization	8.00	7.89	-0.11
Vaccine Doses	1,037,847	1,037,847	0.00
Costs			
GP Consultations	81,739 €	80,663 €	-1,076 €
Hospitalizations due to Influenza	710,476 €	701,447 €	-9,029 €
Deaths due to Influenza Hospitalization	121,553 €	119,977 €	-1,576 €
Hospitalizations due to Pneumonia	128,214 €	126,553 €	-1,661 €
Deaths due to Pneumonia Hospitalization	90,401 €	89,220 €	-1,181 €
Hospitalizations due to RD	99,127 €	97,877 €	-1,250 €
Deaths due to RD Hospitalization	2,731 €	2,698 €	-33 €
Hospitalizations due to HD	256,052 €	252,779 €	-3,273 €
Deaths due to HD Hospitalization	62,303 €	61,493 €	-810 €
Vaccine Doses	2,673,494 €	7,983,576 €	5,310,082 €
Total	8,029,692 €	13,323,148 €	5,293,456 €
CI lower bound	6,904,148 €	11,292,400 €	4,375,217 €
CI upper bound	9,018,064 €	15,125,063 €	6,088,637 €
QALYs			
Total	1,265,457.89	1,265,458.09	0.20
CI lower bound	1,197,368.63	1,197,368.75	0.06
CI upper bound	1,328,396.10	1,328,396.36	0.43
ICER (€/QALY)			34,501,793
CI lower bound			13,052,665
CI upper bound			87,418,632
