# Supplementary figures and images for: Cost Effectiveness of Quadrivalent Versus Trivalent Inactivated Influenza Vaccines for the Portuguese Elderly Population
Source: Vaccines (Basel). 2022 Aug 9;10(8):1285. doi: 10.3390/vaccines10081285 (PMC9416623; doi:10.3390/vaccines10081285)

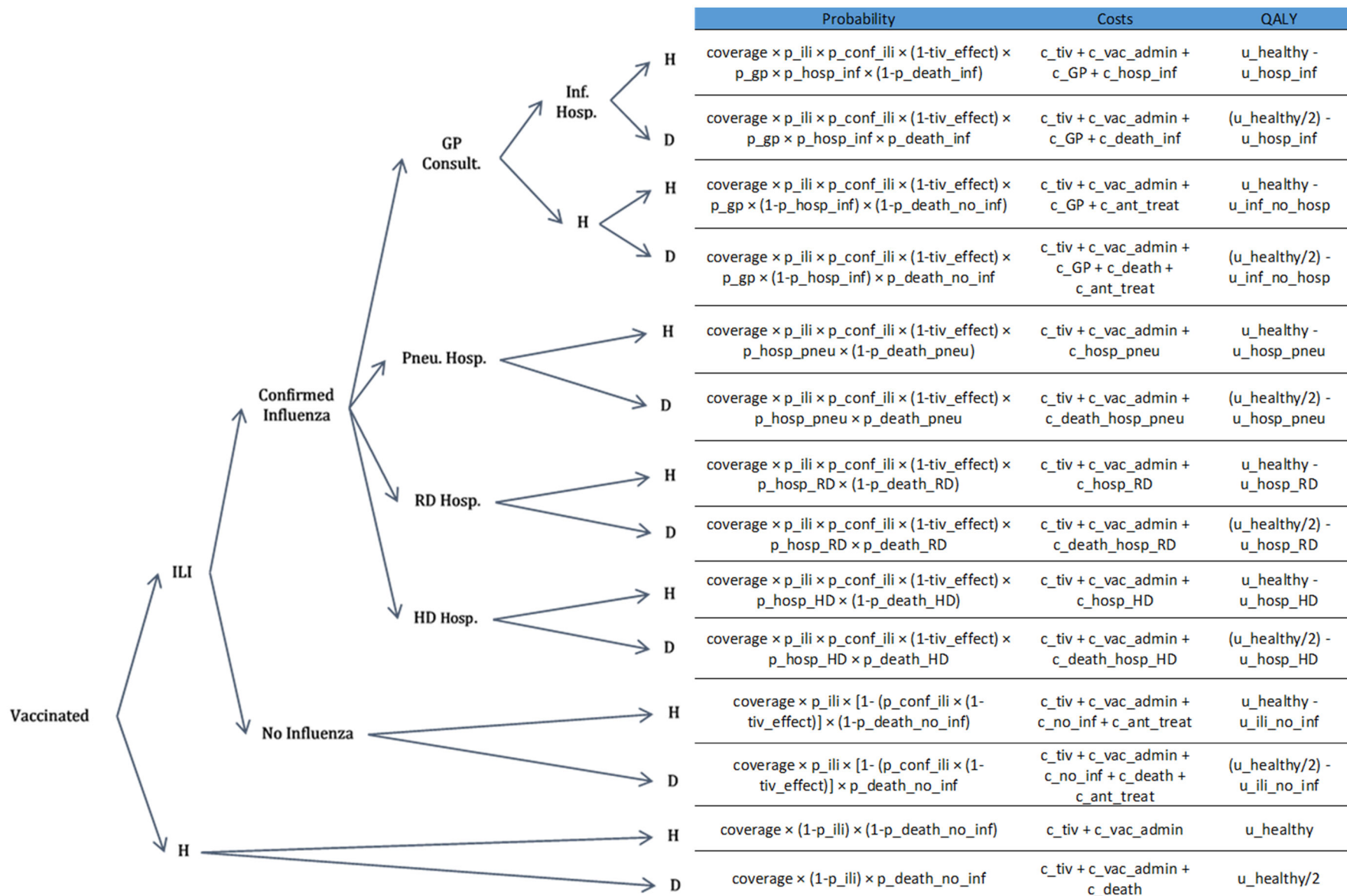

Supplement: Supplementary file 1 [file vaccines-10-01285-s001.zip › Figure S1.pdf]

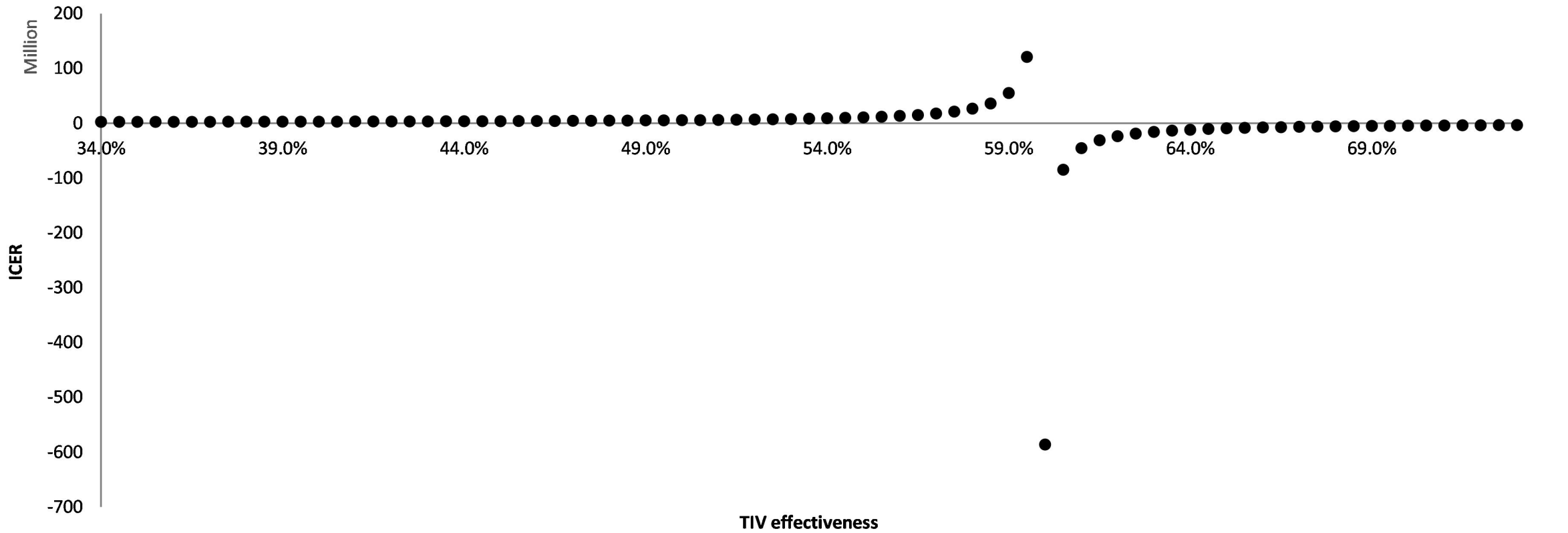

Supplement: Supplementary file 1 [file vaccines-10-01285-s001.zip › Figure S2.pdf]

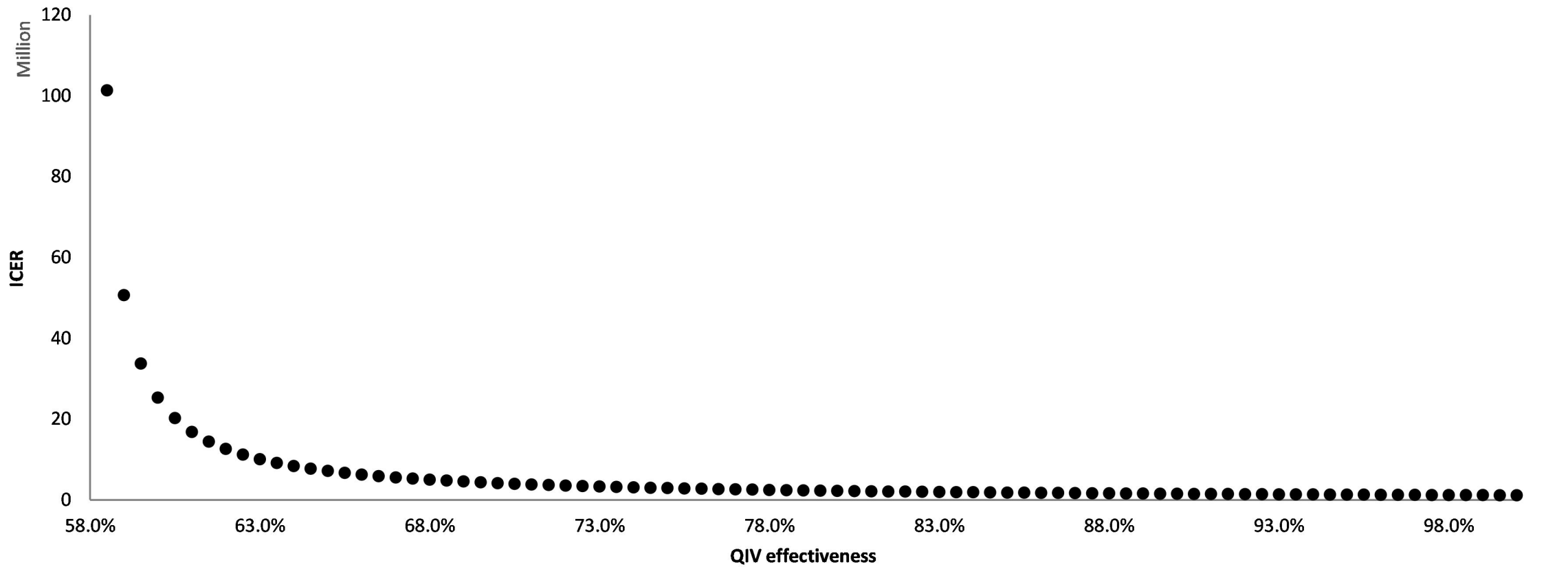

Supplement: Supplementary file 1 [file vaccines-10-01285-s001.zip › Figure S3.pdf]

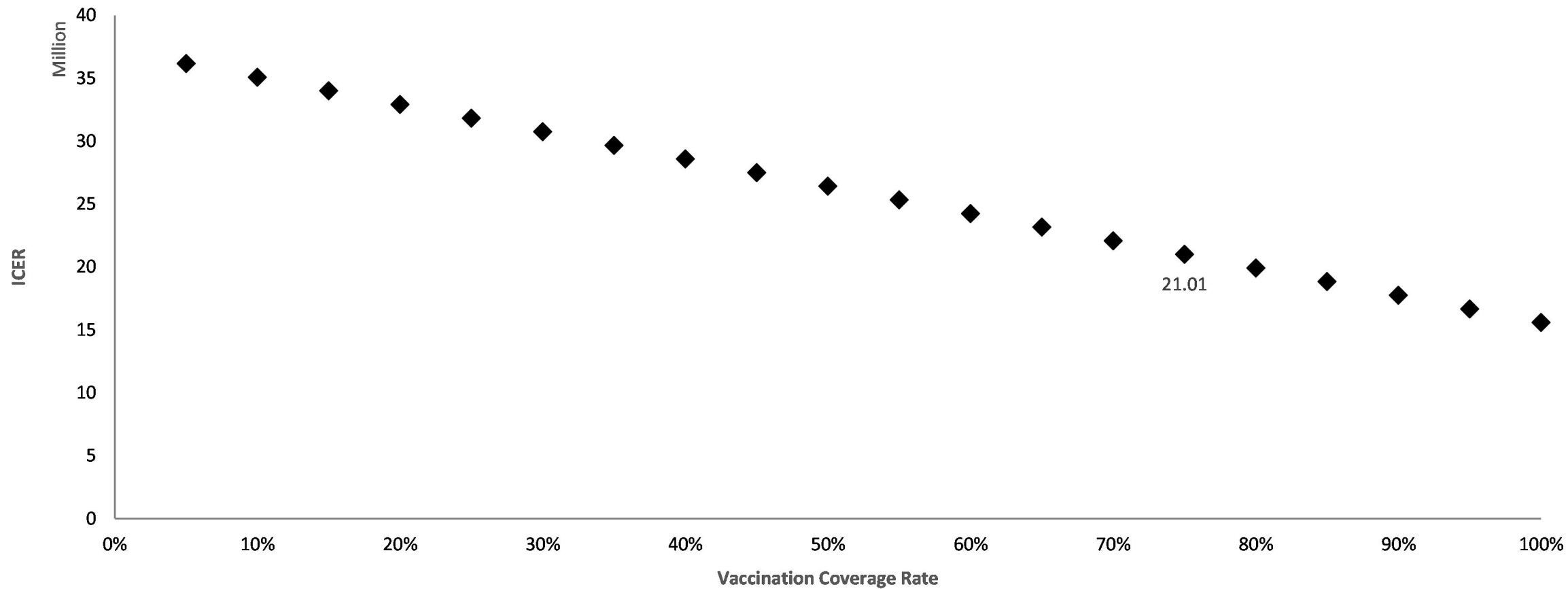

Supplement: Supplementary file 1 [file vaccines-10-01285-s001.zip › Figure S4.pdf]

**The Cost-effectiveness Plane**

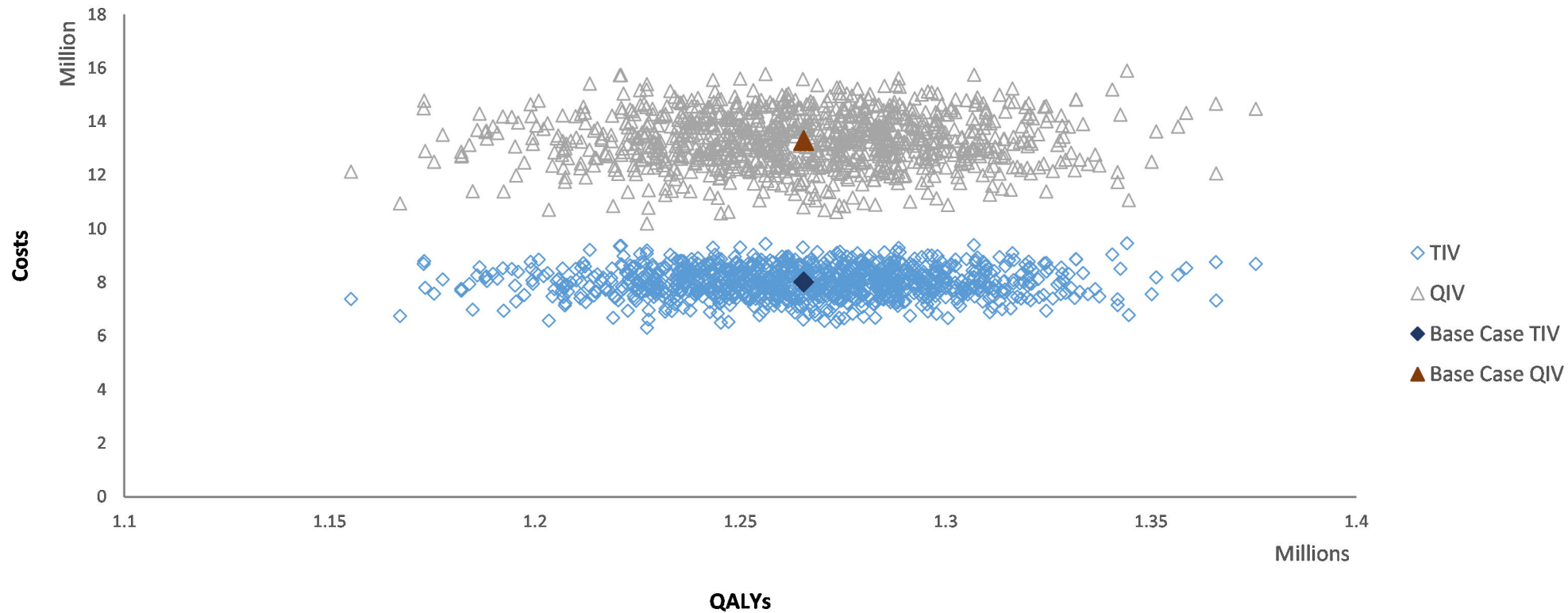

Supplement: Supplementary file 1 [file vaccines-10-01285-s001.zip › Figure S5.pdf]
